# The primary election study 2024 dataset on U.S. Senate primary elections

**DOI:** 10.1016/j.dib.2026.112762

**Published:** 2026-04-09

**Authors:** Sarah E. Anderson, Daniel M. Butler, Peter K. Enns, Laurel Harbridge-Yong, Jacob E. Rothschild

**Affiliations:** a University of California Santa Barbara, Bren School of Environmental Science & Management, 4510 Bren Hall, University of California, Santa Barbara, CA 93106-5131, USA; b Washington University in St. Louis, MSC 1063-228-207, One Brookings Drive, St. Louis, MO 63130-4899, USA; c Cornell University and Verasight, 205 White Hall, Ithaca, NY, 14853, USA; d Northwestern University, Scott Hall, 601 University Place, Evanston, IL 60208, USA; e Verasight, 850A Union Street. San Francisco, CA 94133, USA

**Keywords:** Candidate evaluations, Voter behavior, Ideology, Survey data, Political representation

## Abstract

This article describes the Primary Election Study (PES), a survey dataset designed to facilitate the study of voter behavior, attitudes, and decision-making in U.S. primary elections. The PES was fielded during the 2024 U.S. Senate primary elections in California, Michigan, and Nevada and includes both representative samples of the adult population and large oversamples of likely primary voters. The study collected data from 8124 respondents through a pre-election survey administered in the weeks leading up to each state’s primary contest. The dataset contains detailed measures of candidate evaluations, issue priorities, partisan attitudes, political engagement, and validated turnout, allowing researchers to distinguish between reported voting intentions and verified participation. Survey weights are provided to support analyses of statewide populations, likely primary electorates, and combined samples. The PES addresses limitations of existing election surveys by capturing voter preferences contemporaneously with primary elections and by ensuring adequate statistical power for analyses of primary electorates. These data are suitable for research on electoral behavior, political polarization, representation, and democratic accountability, as well as for teaching and methodological replication.

Specifications Table

**Table:** 

Subject	Social Sciences
Specific subject area	Elections and voting behavior
Type of data	Survey data in csv format. Gives raw responses.
Data collection	Pre-election survey administered online using registration-based sampling and online recruitment
Data source location	United States (California, Michigan, Nevada)
Data accessibility	Repository name: The Harvard Dataverse Dataverse for all 2024 Primary Election Study Data: https://dataverse.harvard.edu/dataverse/PrimaryElectionStudy Data files are shared in two components – Common Content and WUSTL Team Content Common Content: Data identification number: 10.7910/DVN/K1VPKS Direct URL to data: https://dataverse.harvard.edu/dataset.xhtml?persistentId=doi:10.7910/DVN/K1VPKS WUSTL Team Content: Data Identification number: 10.7910/DVN/4XBCEV Direct URL to data: https://dataverse.harvard.edu/dataset.xhtml?persistentId=doi:10.7910/DVN/4XBCEV
Related research article	None

## 1 Value of the Data

• These data provide contemporaneous measures of voter attitudes and preferences during U.S. Senate primary elections, reducing recall bias common in post-election surveys.
• Large oversamples of likely primary voters enable precise analysis of primary electorates that are typically underrepresented in general election surveys.
• The inclusion of validated turnout data allows researchers to distinguish between actual voters and non-voters, improving measurement accuracy.
• The dataset supports comparative analyses between primary electorates and the broader adult population, facilitating research on representation and accountability.

## 2 Background

Primary elections play a critical role in the U.S. electoral process by shaping candidate selection and influencing subsequent legislative behavior [[1], [2], [3], [4], [5], [6], [7]]. Despite their importance, empirical research on primary electorates has been constrained by data limitations, including small sample sizes and retrospective measurement. The Primary Election Study (PES) was designed to address these limitations by collecting large-scale survey data during active primary election periods [8]. The dataset provides a foundation for studying how primary voters evaluate candidates, prioritize issues, and engage with the electoral process.

## 3 Data Description

The PES dataset includes survey responses from 8124 individuals across California, Michigan, and Nevada. Each state sample consists of a representative adult population sample and oversamples of likely primary voters aligned with competitive party contests. The dataset contains respondent-level variables capturing demographics, partisan identification, issue positions, candidate evaluations, political engagement, and validated primary election turnout. Accompanying documentation includes a codebook and survey weights designed for population and electorate-level inference.

## 4 Experimental Design, Materials and Methods

During the 2024 primary season, we led a group of five separate teams that surveyed representative state samples for the U.S. Senate primary elections in California, Nevada, and Michigan (1000 each). These samples were designed to be representative of the resident population aged 18 and older in each state. California has an all-candidate, top-two primary while Michigan and Nevada have partisan primaries. Given the primary races that were anticipated to be competitive, we over-sampled 1000 Democratic primary voters in California^1^ and Michigan and 1000 Republican primary voters in California, Michigan, and Nevada. These samples were designed to be representative of primary voter population in each state. The survey was conducted as a 15-minute pre-election survey by Verasight, half common content and half randomized to one of five team modules. This yielded a sample of 8124 respondents:

• **California**: 3117 respondents (N = 1000 general population, 1055 likely Republican primary voters, 1062 likely Democratic primary voters, fielded January 23-March 5, 2024)
• **Nevada**: 2004 respondents (N = 1004 general population, 1000 likely Republican primary voters, fielded May 1-June 11, 2024)
• **Michigan**: 3003 respondents (N = 1001 general population, 1002 likely Republican primary voters, 1000 likely Democratic primary voters, fielded June 25-August 6, 2024)

Data collection was managed by Verasight, which employed a multi-mode technique to ensure representativeness and reach specific primary electorates:

1. **Registration-Based Sampling (RBS)**: Uses official voter records to target representative state samples and individuals with a history of primary participation. This accounted for 74.6% to 89.2% of the samples across the three states. These respondents were recruited via a combination of physical mail and text messages, both of which included a link to a web-based survey.
2. **Dynamic Online Targeting**: Utilized geolocation and advertisements on social media and search engines to reach respondents within target states. These respondents were recruited online and directed to a web-based survey.
3. **Verasight Community**: A pool of verified survey takers initially empaneled via a combination of random address-based sampling, random person-to-person text messaging, and dynamic online targeting. Verasight’s dynamic online targeting reaches potential respondents with customized online invitations to join the Verasight Community. Verification occurs at panel sign up via text message confirmation through a major US phone carrier, during each survey via a variety of data quality controls, and across surveys through Verasight’s passive verification system, which ensures internally consistent responses. For recruitment into the PES sample, Verasight Community members were contacted via email and completed the survey on the web.

### 4.1 Data quality and validation

To ensure high-quality data, all Verasight Community members are verified via multi-step authentication; those who exhibit low-quality response behaviors over time, such as straight-lining or speeding, are removed and prohibited from further participation in the Community. During data cleaning and validation, samples from each source are first combined and then deduplicated using IP addresses, contact information, and in-survey metadata to ensure each survey response represents a unique individual.

The data also include validated vote (i.e., did respondents actually participate in the 2024 primary elections) for most respondents (87.3% in California, 88.2% in Nevada, and 96.8% in Michigan), which highlights the success at recruiting both a general population sample that includes likely and unlikely voters and an oversample of actual primary voters. For instance, among California respondents the PES classified as likely voters, 75% voted, compared to 17% who did not and 8% whose identity we could not match to the validated voter files. Although the precision of estimates for unlikely voters is lower (as in other election studies like the Cooperative Election Study because it groups together those who are very unlikely to vote with those who think they might vote), this subgroup was more likely not to vote (39%) than to vote (31%). In short, the validated vote indicates that the data identifies actual primary voters, not just social desirability to report an intent to vote. As a result, PES data, either as full data or as the subset of validated vote respondents, can be used to study primary voters alone via the oversample or to compare primary voters to the general population.

### 4.2 Weighting

For each state, PES data include survey weights that allow researchers to make the data comparable to state demographics and/or to the distribution of primary voters in each state. The study provides the following survey weights:

• weights_genpop: Can be applied to the general population sample to replicate the statewide adult population
• weights_lv: Can be applied to the likely primary voter oversamples to replicate the likely primary voter population in each state.
• weights_lv_full: Can be applied to all likely primary voters (from both the likely primary voter oversample and the general population samples).
• weight_comb: Can be applied to the full data to replicate the statewide adult population. However, because our oversamples of likely primary voters are so large, this weight has a larger design effect.

**Variables in the Data:**

The text below provides an overview of the sections and the questions asked in the common content and the WUSTL team module of the PES. Below each description of the section, we provide example questions. A complete codebook is available on the Dataverse. Team content for the other four teams will be added to the Dataverse in early-fall 2026.

### 4.3 Common content – eligibility

These questions capture respondent’s eligibility for the survey, including their informed consent, the year they were born (age), and their state of residence.

• Age: Please select the year you were born [drop-down with years]

### 4.4 Common content – electoral participation

These questions characterize whether respondents are registered to vote, their likelihood of participating in the upcoming primary election, the party of the primary they will vote in (or would vote in if they opted to vote), and likelihood of participating in the November general election. Because CA has a non-partisan primary, respondents in CA are asked which party’s primary they would vote in under the old partisan system. The principal investigators developed these questions for this project.

• Primary likelihood: Rate your chance of voting in the upcoming primary election in your state on a scale of 1 to 10, with 1 being “definitely will not vote” and 10 “definitely will vote.” If you have already voted early or absentee, select 10.
• Party of primary (if likely to vote): In which party’s primary will you vote? Democratic Party, Republican Party, Other
• Party of primary (if not likely to vote): If you were to vote, in which party’s primary would you vote? Democratic Party, Republican Party, Other.

### 4.5 Common content – demographics

These questions characterize respondent’s demographics: gender, education, race and ethnicity, income, religion, and religious attendance. These questions follow similar demographic questions in the American National Election Study [9] and the Cooperative Election Study [10].

• Gender: How would you describe your gender? Woman, Man, Other, Prefer not to say

### 4.6 Common content – partisanship and ideology

These questions characterize respondent’s partisanship using the branching approach to create a 7-point party identification measure. They also ask respondents to place themselves, the national party leaders (Donald Trump and Joe Biden), and several candidates for the Senate primary on a 7-point liberal-conservative scale. For partisan primaries, the candidates are from the party the respondent selected for primary participation; for the non-partisan primary, the candidates are the leading candidates from across both parties. The party identification and ideological placement questions follow similar questions in the American National Election Study [9] and the Cooperative Election Study [10]. The principal investigators for this project identified which Senate primary candidates to include based on polling and/or fundraising reports to assess viability.

• Ideology: Please rate the following individuals in terms of their ideology
  ○ Yourself
  ○ Donald Trump
  ○ Joe Biden
  ○ [Candidates for office]

### 4.7 Common content – electability

Assesses perceived electability of the candidates (i.e., how the party would do in the general election if that candidate were the party’s nominee). The electability questions draw on the wording used by Anderson et al. [11]. The principal investigators for this project identified which Senate primary candidates to include based on polling and/or fundraising reports to assess viability.

• Electability: For each of the [Democratic/ Republican] candidates below, what is the chance that the [Democrats/Republicans] will win November’s general election Senate race in [STATE] if that candidate is on the ballot in November? [0–100 scale].

### 4.8 Common content – voice choice

These questions ask respondents which candidate they would vote for in the Senate primary, as well as which Presidential primary candidate they would vote for. There is also an open-ended question asking for the main reason respondents picked their candidate in the Senate primary. These questions were written by the principal investigators for this project. Note: For states with partisan primaries, the Senate primary choices reflect all candidates on the ballot of the party the respondent selected for primary participation. For the non-partisan primary in CA, the Senate primary choices reflect all candidates on the ballot, regardless of party.

• Senate primary vote [if MI/NV]: If you were voting today in the [STATE] [Republican/Democratic] primary, which candidate for the US Senate would you vote for?
• Senate primary vote [if CA]: If you were voting today in the California primary, which candidate for the US Senate would you vote for?

### 4.9 Common content – feeling thermometers

These questions characterize how warm or cold respondents feel toward the Democratic and Republican parties. These questions are commonly used for measuring affective polarization [12] and follow similar feeling thermometer questions in the American National Election Study [9] and the Cooperative Election Study [10].

• Feeling Thermometers: Below is a “thermometer scale” where 0 means “very cold” and 100 means “very warm.” The higher the number, the warmer or more favorable you feel; the lower the number, the cooler or less favorable you feel. Please rate how warm or cold you feel towards each of the following groups.
  ○ Democratic Party
  ○ Republican Party

### 4.10 Team block (WUSTL) – additional respondent traits

These questions provide additional traits of the respondents. These include assessments of the importance of partisanship, religion, gender, and race to their identity; how closely they follow politics; hostile sexism [13]; and racial resentment. These questions build upon similar questions in other studies, including the American National Election Study [9] and the Cooperative Election Study [10].

• Identity importance: How important are each of the following to your own identity? [Your partisanship, Your religion; Your gender; Your race]
• Sexism: Please indicate the degree to which you agree or disagree with each statement: Women seek to gain power by getting control over men.
• Racial resentment: Please indicate the degree to which you agree or disagree with each statement: Racial problems in the U.S. are rare, isolated incidents.

### 4.11 Team block (WUSTL) – support for undemocratic practices

These questions capture respondent’s willingness to support actions that violate democratic norms. Respondents are also asked about their perceptions of how voters from the other party think about political violence. These questions draw on those used in the Stanford Strengthening Democracy Challenge [14].

• Support for undemocratic practices: Please indicate how much you agree or disagree with each of the following statements.
  ○ [Democratic/Republican] governors should ignore unfavorable court rulings by [Republican/Democratic]-appointed judges.
  ○ [Democrats/Republicans] should not accept the results of elections if they lose.
• How do you think the average [Democratic/Republican] voter would answer this question: When, if ever, is it OK for [Democrats/Republicans] to send threatening and intimidating messaging to [Republican/Democratic] party leaders?

### 4.12 Team block (WUSTL) – party factions

These questions were developed by the principal investigators to capture the respondents’ identification within their party and their perceptions of the party’s direction.

• Faction: Within the [Democratic/Republican] Party, there are different groups of voters. Which of these labels best describes you: [factions of party listed]
• Right track: All in all, do you think, the [Democratic/Republican] Party is on the right track or wrong track?

### 4.13 Team block (WUSTL) – community perceptions and interactions

These questions assess respondents’ perceptions of their community, respondents’ own views on whether voting is a duty or choice, and their frequency of conversations about the primary election. They include items developed by the principal investigators on community attitudes toward the Republican and Democratic parties, as well as views about voting as a civic duty in both primary and general election contexts (simplified from the ANES [9]). In developing the questions about discussing the Senate primary with others, the principal investigators also consulted with Dr. Lisa Argyle.

• Community feeling thermometers: How do people in your community feel about the Democratic and Republican Party using a scale of 0 to 100?
• Frequency of conversations: How many days in the last week have you discussed the [STATE] primary election for the U.S. Senate with the people around you?

### 4.14 Team block (WUSTL) – individual interactions with others

These questions focus on how respondents interact with small groups of people, which draw on questions on self-monitoring [[15], [16]] and questions about whether they think they could keep their vote in the primary secret, which were inspired by [17].

• Vote secret: Suppose you wanted to keep the party you voted for in the primary secret from your close friends. Do you think you could keep them from finding out or would you eventually say something that allowed them to figure out which party’s primary you voted in?

### 4.15 Team block (WUSTL) – issue and endorsements

These questions ask about salient political issues. This includes the respondents’ own positions and their perceptions about candidates’ positions. It also asks if groups they belong to have endorsed any candidates in the Senate rate. These questions were developed by the principal investigators for this project. We also included a question used in past Pew surveys about respondents’ preferences about candidates’ willingness to compromise [18].

• Candidate issue agreement: On the issue of [XX], do you think that each of the candidates below agree or disagree with your position?
• Compromise: Which comes closest to your own views – even if neither is exactly right? I like elected officials who make compromises with people they disagree with. I like elected officials who stick to their positions

## Limitations

Not applicable

## Ethics Statement

All procedures involving human participants were reviewed and approved by the relevant Institutional Review Board (Study #: 202310086). Informed consent was obtained from all participants prior to data collection.

## CRediT authorship contribution statement

**Sarah E. Anderson:** Conceptualization, Funding acquisition, Writing – original draft. **Daniel M. Butler:** Conceptualization, Funding acquisition, Writing – original draft. **Peter K. Enns:** Methodology, Writing – review & editing. **Laurel Harbridge-Yong:** Conceptualization, Funding acquisition, Writing – original draft. **Jacob E. Rothschild:** Methodology, Data curation, Validation.

## Data Availability

Dataverse2024 Primary Election Study (Original data).

## Footnotes

1 For California, oversamples of likely primary voters are separated by party even though the state holds a non-partisan top-2 primary.
